# Reminiscence therapy-based care program alleviates anxiety and depression, as well as improves the quality of life in recurrent gastric cancer patients

**DOI:** 10.3389/fpsyg.2023.1133470

**Published:** 2023-06-07

**Authors:** Xing Wu, Weiwei Zhang

**Affiliations:** ^1^Department of General Surgery, HanDan Central Hospital, Handan, China; ^2^Department of Hematology, HanDan Central Hospital, Handan, China

**Keywords:** recurrent gastric cancer, reminiscence therapy-based care program, anxiety, depression, quality of life

## Abstract

**Objective:**

Reminiscence therapy is a non-drug method that eases psychological burden and enhances quality of life by memories and communications in cancer patients. This study aimed to evaluate influence of reminiscence therapy-based care program on anxiety, depression, and quality of life in recurrent gastric cancer patients.

**Methods:**

Totally, 96 recurrent gastric cancer patients were randomly assigned as 1:1 ratio into reminiscence therapy-based care group (*N* = 48) and usual care group (*N* = 48) to receive 12-week corresponding interventions. Besides, all patients were follow-up for 6 months.

**Results:**

Hospital Anxiety and Depression Scales-anxiety score at 4th month (*p* = 0.031) and 6th month (*p* = 0.004), Hospital Anxiety and Depression Scales-depression score at 6th month (*p* = 0.018), and anxiety severity at 4th month (*p* = 0.041) and 6th month (*p* = 0.037) were lower in reminiscence therapy-based care group than in usual care group. Quality of Life Questionnaire-Core 30 global health status score at 2nd month (*p* = 0.048), 4th month (*p* = 0.036), and 6th month (*p* = 0.014), Quality of Life Questionnaire-Core 30 function score at 4th month (*p* = 0.014) and 6th month (*p* = 0.021) were higher, while Quality of Life Questionnaire-Core 30 symptoms score at 2nd month (*p* = 0.041) and 4th month (*p* = 0.035) were lower in reminiscence therapy-based care group than in usual care group. Furthermore, reminiscence therapy-based care was more effective on improving mental health and quality of life in recurrent gastric cancer patients with anxiety or depression at baseline than those without.

**Conclusion:**

Reminiscence therapy-based care serves as an effective intervention, which relieves anxiety and depression, and improves quality of life in recurrent gastric cancer patients.

## 1. Introduction

Gastric cancer (GC) has high morbidity and mortality globally, which causes more than one million new cases and over 700,000 new deaths in 2020 (Thrift and El-Serag, 2020; Sung et al., 2021). Moreover, the risk factors for GC include family history, poor diet, alcohol, etc. (Machlowska et al., 2020). Meanwhile, the mainstay of GC treatment is surgical resection, and other treatments include chemotherapy, targeted drug therapy, immunotherapy and so on (Machlowska et al., 2020; Sexton et al., 2020; Joshi and Badgwell, 2021). Although advances in diagnosis and treatment modalities have been made to increase the survival of patients, GC patients still face a high risk of recurrence (Moon et al., 2007; de Liano et al., 2008; Kong et al., 2015; Jiao et al., 2020). Due to a series of adverse physiological reactions caused by long-term illness and treatments, recurrent GC patients usually have a huge psychological burden, which could induce anxiety and depression (Han, 2020; Zhang, 2021). In addition, their quality of life is also unsatisfactory, which may lead to deterioration of those patients’ conditions and even death (Zieren et al., 1998). Therefore, how to alleviate the anxiety and depression, as well as enhance quality of life in recurrent GC patients is a matter of concern.

Reminiscence therapy (RT) is a non-drug intervention therapy that guides people to review past memories and share life experiences under some tangible cues (such as photos, music, and recordings), it reduces negative reminiscence and increases positive reminiscence, thus alleviating mental health and improving quality of life in patients (Macleod et al., 2021; Sun et al., 2023). A previous study shows that RT is a prospective nursing modality to relieve the anxiety and depression of glioma patients (Zhao, 2021). Moreover, other researchers suggest that RT can also relieve the anxiety and depression and enhance quality of life in postoperative patients with non-small cell lung cancer, surgical prostate cancer, colorectal cancer, etc. (Liu and Li, 2021; Zhou and Sun, 2021; Huang et al., 2022). In addition, one study reports that RT eases anxiety and enhances quality of life in postoperative new-diagnosed GC patients (Zhang et al., 2021). The above studies exhibit the potential of RT as an intervention to alleviate anxiety and depression, as well as improve quality of life in cancer patients, however, the effect of RT on these aspects for recurrent GC patients is still unidentified.

Therefore, the current study was to compare the effect of RT-based care (RTC) program with usual care (UC) program on anxiety, depression, and quality of life in recurrent GC patients.

## 2. Methods

### 2.1. Participants

In this randomized, controlled trial, between Aug. 2019 and Oct. 2021, 96 patients with recurrent GC were enrolled. The inclusion criteria were: (Thrift and El-Serag, 2020) patients with age older than 18 years; (Sung et al., 2021) patients with recurrent GC; (Machlowska et al., 2020) patients who were able to complete the assessment independently; (Sexton et al., 2020) patients who were willing to communicate with others. The exclusion criteria were: (Thrift and El-Serag, 2020) patients complicated with primary malignancies other than GC; (Sung et al., 2021) patients with neurological diseases, cognitive dysfunction, or mental illness; (Machlowska et al., 2020) patients without the capability of normal communication. The Institution Review Board of HanDan Central Hospital approved this trial. Written informed consent were obtained from all patients.

### 2.2. Randomization

After enrollment, patients were randomly assigned to receive UC program (UC group) and RTC program (RTC group). The block randomization method was applied to propose a random allocation table with a block size of 4 to achieve a 1:1 random assignment. Then, the random allocation information of each patient was closured in an opaque wrapper, corresponding to the enrollment series number of the patient. Based on that, the opaque wrappers were given to the eligible patients and then the participants were allocated to the corresponding group.

### 2.3. Intervention

Based on the grouping, patients received UC or RTC program. The interventions were performed in the group form (8–10 patients per group) in the health care center of our hospital every week for 12 weeks.

Patients in the UC group received health education after enrollment, which included an outline of recurrent GC, treatment, adverse events and management, examinations, self-monitoring, diet and lifestyle, and psychological health. During the UC program, the multimedia information and communication technology such as tablet personal computer and large-screened monitors were used as needed. Besides, the health promotion brochures were distributed at the same time and available for patients to consult at any time. UC was lasted for 30 min each time. Two trained nurses hosted UC.

Patients in the RTC group received RTC at our hospital. RTC was constituted with two parts: (i) health education, which was the same as that in the UC group and (ii) RT. RT was performed in group and on the basis of 12 topics: (Thrift and El-Serag, 2020) self-introduction and a brief outline of your family; (Sung et al., 2021) sharing childhood memories; (Machlowska et al., 2020) sharing campus life; (Sexton et al., 2020) sharing memories of marriage (memories of love for patients not married); (Joshi and Badgwell, 2021) sharing unique traditions of your homeland; (Jiao et al., 2020) sharing the stories in your career (the stories of teamwork for patients who had not been employed); (Moon et al., 2007) sharing a memorable travel experience; (de Liano et al., 2008) sharing your best-loved movie or songs; (Kong et al., 2015) sharing your personal leisure pursuit; (Zhang, 2021) sharing your best-loved historical figure and their well-known legend; (Han, 2020) talent show; (Zieren et al., 1998) review and summarization. During the RTC program, the multimedia information and communication technology were also used, and the health promotion brochures were distributed as well. The duration of each RTC was 100 min, including 30 min of health education, 10 min of break, and 60 min of RT. Two trained nurses hosted the RTC, motivated the patients to communicate, and kept the whole procedure in order.

### 2.4. Evaluation

At baseline (M0), 1st month (M1), 2nd month (M2), 4th month (M4), and 6th month (M6), Hospital Anxiety and Depression Scales (HADS) and Quality of Life Questionnaire-Core 30 (QLQ-C30) were assessed (Aaronson et al., 1993; Wu et al., 2021). Anxiety and depression were considered to exist if HADS-anxiety (HADS-A)/HADS-depression (HADS-D) score > 7; and the severity of anxiety and depression was divided based on HADS-A/HADS-D score as follows: <7, no; 7–10, mild, 11–14, moderate; >14, severe. QLQ-C30 included global health status score, function score, and symptoms scores.

### 2.5. Sample size calculation

The size of sample was reckoned on the basis of that the mean QLQ-C30 Global health status at M6 was hypothesized to be 75 (standard deviation (SD) = 25) in the RTC group, and 60 (SD = 20) in the UC group (Li et al., 2022). The significance level was set as 0.05, and the power was set as 0.8. Therefore, the minimal sample size was required to be 35 in each group. Given that 25% patients may lost to follow-up or die during 6 months, the final size of sample was required to be 48 in each group.

### 2.6. Statistics

SPSS (22.0, IBM) and Graphpad Prism (6.01, GraphPad Software Inc.) was adopted for data analyses and figure illustration, accordingly. The intention-to-treat (ITT) principal was adopted in this study. Student’s t-test, Chi-square test, or Wilcoxon rank-sum test was utilized to compare variables between groups. Trend within group was determined using repeated measures analysis of variance (ANOVA), McNemar’s test, or Friedman’s test. Statistical significance was considered if a *p* value<0.05.

## 3. Results

### 3.1. Study flow

**Figure 1:**
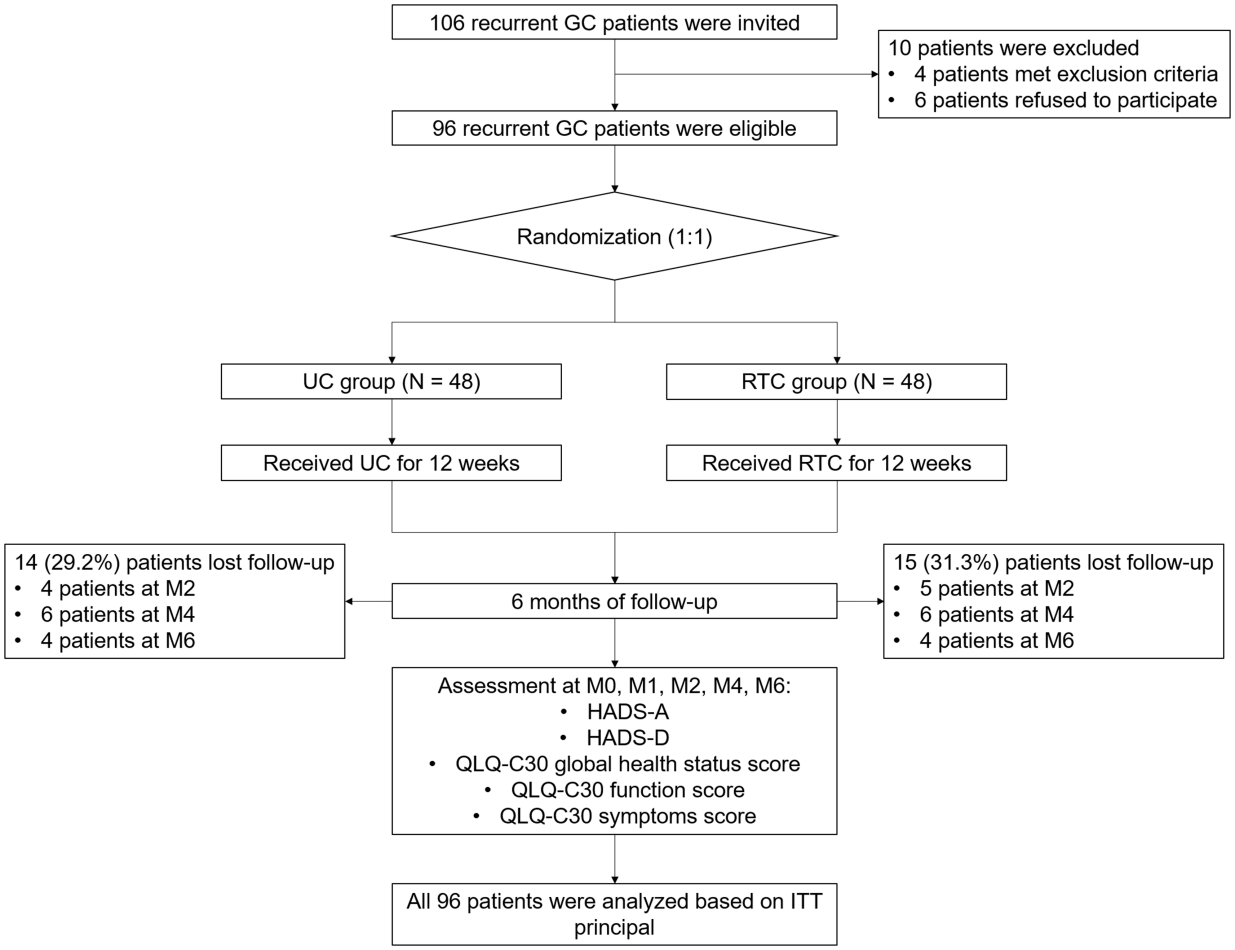
Study flow chart.

In total, 106 recurrent GC patients were invited, 10 of whom were excluded from this study, including 4 patients who fit the exclusion criteria or did not fit the inclusion criteria, and 6 patients who refused to participate. Next, the rest of 96 patients were eligible and randomly assigned as 1:1 ratio into UC group (*N* = 48) and RTC group (*N* = 48) to receive UC and RTC interventions for 12 weeks, respectively. During 6-month follow-up period, there were 14 (29.2%) patients who lost follow-up in the UC group, including 4 patients at M2, 6 patients at M4, and 4 patients at M6. Meanwhile, there were 15 (31.3%) patients losing follow-up in the RTC group, including 5 patients at M2, 6 patients at M4, and 4 patients at M6. In addition, HADS scores and QLQ-C30 scores were appraised at M0, M1, M2, M4 and M6, respectively. All 96 patients were analyzed based on ITT principle (Figure 1).

### 3.2. Baseline features of UC group and RTC group

The UC group included 35 (72.9%) males and 13 (27.1%) females, whose mean age was 57.4 ± 11.9 years. Moreover, the RTC group included 29 (60.4%) males and 19 (39.6%) females, whose mean age was 60.9 ± 10.7 years. Meanwhile, there was no discrepancy in baseline features between groups, including demographics, medical histories, disease information at diagnosis and at recurrence, treatment information for recurrence, and baseline HADS and QLQ-C30 scores (all *p* > 0.05) (Table 1).

**Table 1:** Baseline characteristics.

	UC group (*N* = 48)	RTC group (*N* = 48)	*p* value
*Demographics*			
Age (years), mean ± SD	57.4 ± 11.9	60.9 ± 10.7	0.138
Gender, *n* (%)			0.194
Male	35 (72.9)	29 (60.4)	
Female	13 (27.1)	19 (39.6)	
Education duration (years), mean ± SD	9.9 ± 3.6	10.6 ± 4.2	0.331
Marital status, *n* (%)			0.346
Married	38 (79.2)	34 (70.8)	
Single/divorced/widowed	10 (20.8)	14 (29.2)	
Employment status, *n* (%)			0.805
Employed	10 (20.8)	11 (22.9)	
Unemployed	38 (79.2)	37 (77.1)	
History of smoke, *n* (%)	14 (29.2)	18 (37.5)	0.386
History of drink, *n* (%)	18 (37.5)	22 (45.8)	0.408
*Medical histories*			
History of hypertension, *n* (%)	22 (45.8)	23 (47.9)	0.838
History of hyperlipidemia, *n* (%)	10 (20.8)	14 (29.2)	0.346
History of diabetes, *n* (%)	6 (12.5)	9 (18.8)	0.399
*H.pylori* infection, *n* (%)	28 (58.3)	21 (43.8)	0.153
*Disease information at diagnosis*			
Tumor location at diagnosis, *n* (%)			0.340
Cardia	16 (33.3)	20 (41.7)	
Gastric body	22 (45.8)	15 (31.3)	
Gastric antrum	10 (20.8)	13 (27.1)	
Pathological grade at diagnosis, *n* (%)			0.090
G1	5 (10.4)	9 (18.8)	
G2	24 (50.0)	27 (56.3)	
G3	19 (39.6)	12 (25.0)	
Tumor size at diagnosis (cm), mean ± SD	3.5 ± 1.1	3.7 ± 1.2	0.323
T stage at diagnosis, *n* (%)			0.715
1	1 (2.1)	1 (2.1)	
2	2 (4.2)	2 (4.2)	
3	45 (93.8)	44 (91.7)	
4	0 (0.0)	0 (0.0)	
N stage at diagnosis, *n* (%)			0.583
0	13 (27.1)	12 (25.0)	
1	10 (20.8)	15 (31.3)	
2	19 (39.6)	17 (35.4)	
3	6 (12.5)	4 (8.3)	
M stage at diagnosis, *n* (%)			–
0	48 (100.0)	48 (100.0)	
1	0 (0.0)	0 (0.0)	
TNM stage at diagnosis, *n* (%)			0.644
1	3 (6.3)	2 (4.2)	
2	20 (41.7)	24 (50.0)	
3	25 (52.1)	22 (45.8)	
*Disease information at recurrence*			
Time to recurrence, *n* (%)			0.734
<3 years	15 (31.3)	11 (22.9)	
3–5 years	15 (31.3)	20 (41.7)	
≥5 years	18 (37.5)	17 (35.4)	
Recurrent tumor location, *n* (%)			0.065
Cardia	19 (39.6)	17 (35.4)	
Gastric body	26 (54.2)	30 (41.7)	
Gastric antrum	3 (6.3)	11 (22.9)	
Distance metastases at recurrence, *n* (%)			0.519
No	33 (68.8)	30 (62.5)	
Yes	15 (31.3)	18 (37.5)	
*Treatment information for recurrence*			
Chemotherapy, *n* (%)			–
No	0 (0.0)	0 (0.0)	
Yes	48 (100.0)	48 (100.0)	
Radiotherapy, *n* (%)			0.138
No	27 (56.3)	34 (70.8)	
Yes	21 (43.8)	14 (29.2)	
Targeted drug therapy, *n* (%)			0.670
No	18 (37.5)	16 (33.3)	
Yes	30 (62.5)	32 (66.7)	
ICI treatment, *n* (%)			0.805
No	37 (77.1)	38 (79.2)	
Yes	11 (22.9)	10 (20.8)	
*Baseline assessment*			
HADS-A score, mean ± SD	8.7 ± 3.4	8.2 ± 3.2	0.441
HADS-D score, mean ± SD	7.6 ± 2.9	7.5 ± 2.7	0.858
QLQ-C30 global health status score, mean ± SD	59.9 ± 17.4	61.8 ± 14.9	0.568
QLQ-C30 function score, mean ± SD	55.3 ± 17.7	57.2 ± 16.6	0.578
QLQ-C30 symptoms score, mean ± SD	42.2 ± 18.9	41.2 ± 15.8	0.766

UC, usual care program; RTC, reminiscence therapy-based care program; H.pylori: helicobacter pylori; TNM, tumor, node, metastasis; ICI, immune check-point inhibitor; HADS-A, Hospital Anxiety and Depression Scale-anxiety; HADS-D, Hospital Anxiety and Depression Scale-depression; QLQ-C30, Quality of Life Questionnaire-Core 30; SD, standard deviation. -: Unable to perform statistics.

### 3.3. Comparison of anxiety and depression between groups

**Figure 2:**
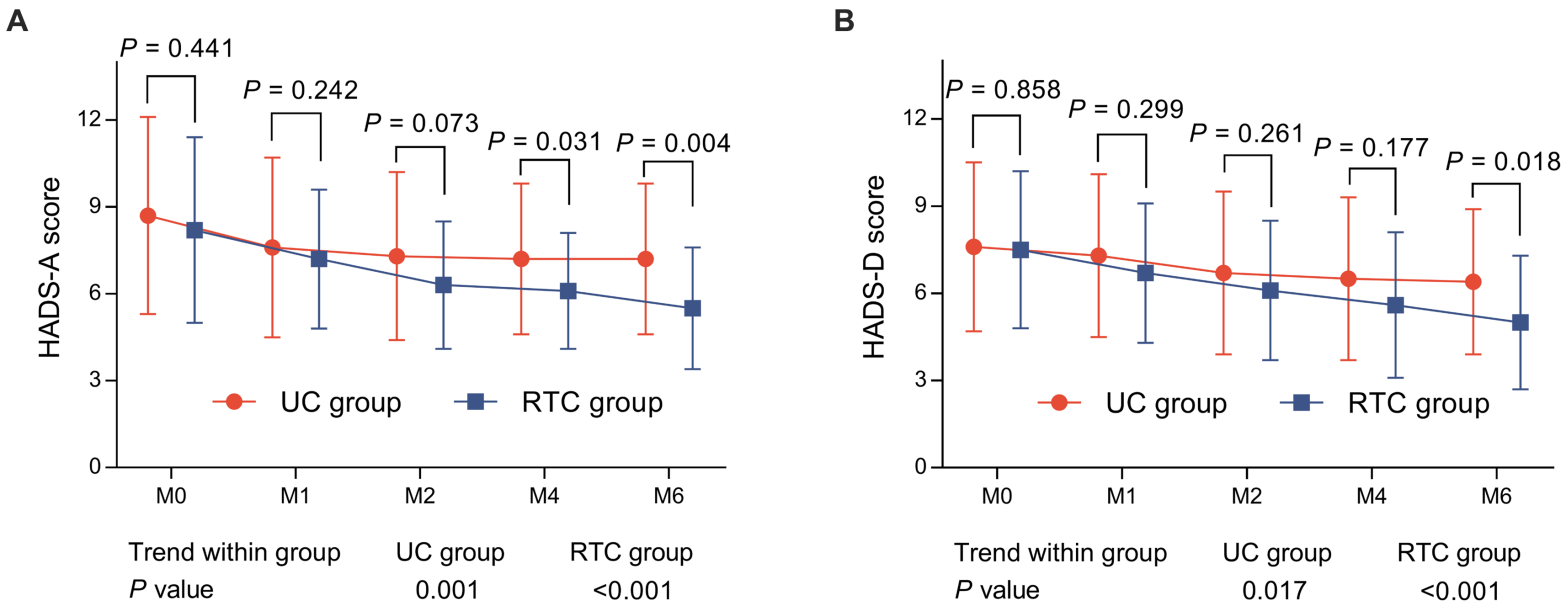
Comparison of HADS scores between groups. HADS-A score **(A)** at M4 and M6, HADS-D score **(B)** at M6 were lower in RTC group than in UC group.

HADS-A score at M4 (6.1 ± 2.0 vs. 7.3 ± 2.6) (*p* = 0.031) and M6 (5.5 ± 2.1 vs. 7.2 ± 2.6) (*p* = 0.004) were lower in RTC group than in UC group. Notably, HADS-A score was gradually reduced from M0 to M6 in RTC group (*p* < 0.001) and UC group (*p* = 0.001), respectively (Figure 2A), moreover, HADS-D score at M6 was lower in RTC group than in UC group (5.0 ± 2.3 vs. 6.4 ± 2.5) (*p* = 0.018), HADS-D score was also gradually decreased from M0 to M6 in RTC group (*p* < 0.001) and UC group (*p* = 0.017), respectively (Figure 2B).

**Figure 3:**
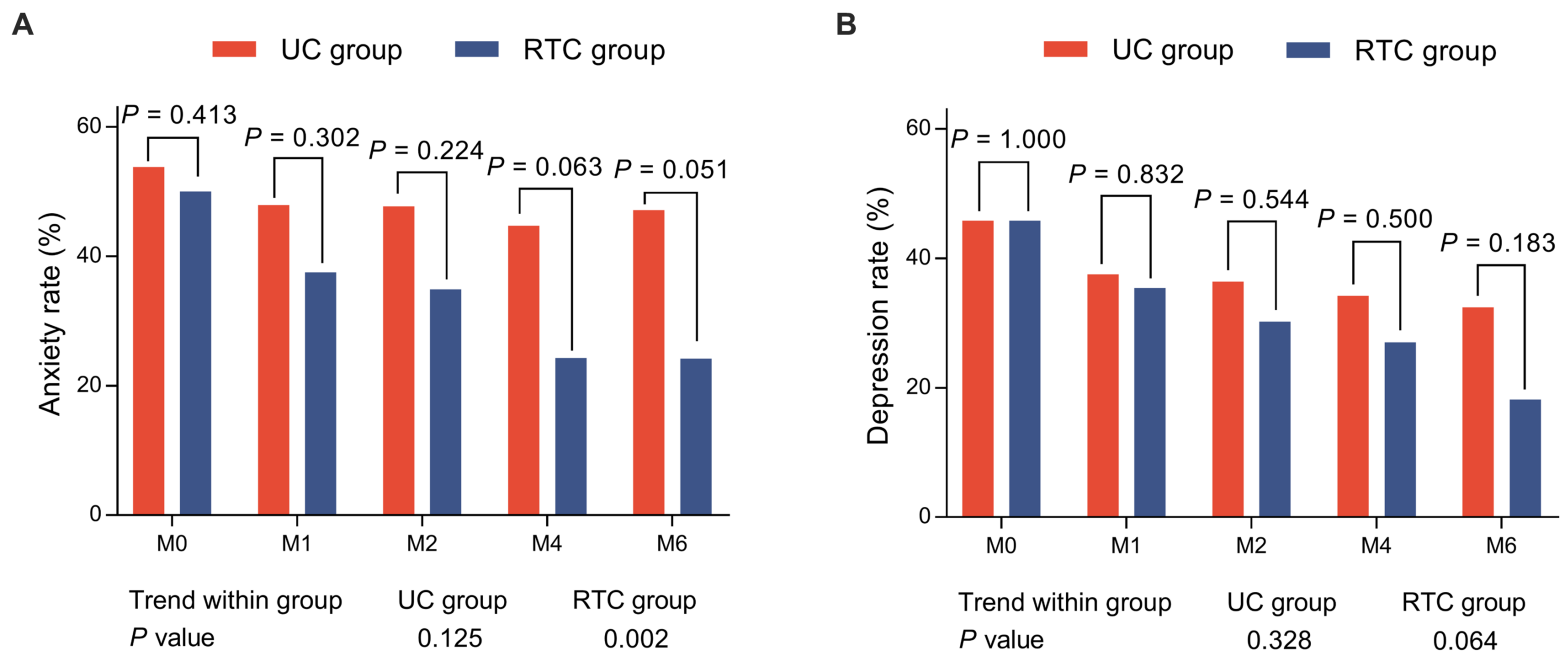
Comparison of anxiety rate and depression rate between groups. Anxiety rate at M6 tended to be lower in RTC group than UC group **(A)**, there was no distinction of depression rate at any assessment time points between groups **(B)**.

Generally, there was no distinction of anxiety rate or depression rate at any assessment time points between groups (all *p* > 0.05). Interestingly, anxiety rate was declined continually from M0 to M6 in RTC group (*p* = 0.002), while it did not change in UC group (*p* = 0.125). Meanwhile, depression rate did not change longitudinally in RTC group (*p* = 0.064) or UC group (*p* = 0.328) (Figures 3A,B). However, anxiety rate at M6 tended to be lower in RTC group than in UC group (*p* = 0.051).

**Figure 4:**
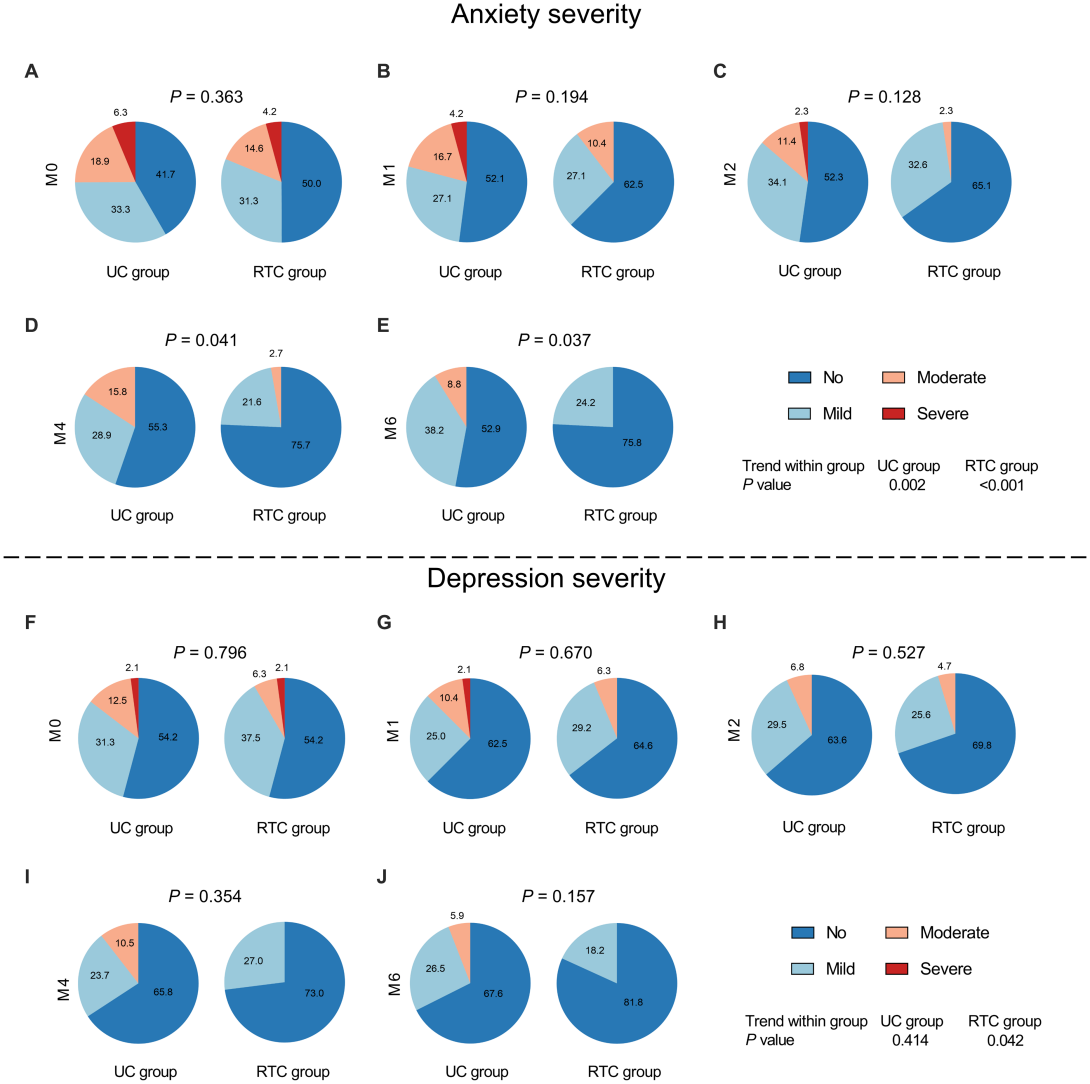
Comparison of anxiety severity and depression severity between groups. There was no difference of anxiety severity at M0 **(A)**, M1 **(B)**, and M2 **(C)** between groups; while anxiety severity at M4 **(D)** and M6 **(E)** was lower in RTC group than in UC group. No difference in depression severity was found at M0 **(F)**, M1 **(G)**, and M2 **(H)**, M4 **(I)**, and M6 **(J)** between groups.

Besides, there was no discrepancy of anxiety severity at M0, M1, or M2 between groups (all *p* > 0.05) (Figures 4A–C), however, anxiety severity at M4 (*p* = 0.041) and M6 (*p* = 0.037) were different between RTC group and UC group. Meanwhile, anxiety severity changed longitudinally in RTC group (*p* < 0.001) and UC group (*p* = 0.002), respectively (Figures 4D–E). Regarding depression severity, no difference was found at any assessment time points between groups (all *p* > 0.05). Moreover, depression severity was changed longitudinally in RTC group (*p* = 0.042), but it did not change in UC group (*p* = 0.414) (Figures 4F–J).

### 3.4. Comparison of QLQ-C30 scores between groups

**Figure 5:**
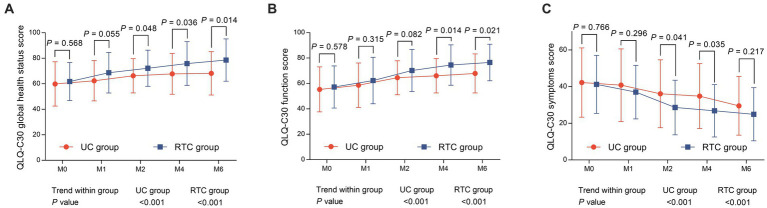
Comparison of QLQ-C30 scores between groups. QLQ-C30 global health status score **(A)** at M2, M4, and M6, and QLQ-C30 function score **(B)** at M4 and M6 were higher; while QLQ-C30 symptoms score **(C)** at M2 and M4 were lower in RTC group than in UC group.

QLQ-C30 global health status score at M2 (72.2 ± 14.2 vs. 66.3 ± 13.4) (*p* = 0.048), M4 (75.9 ± 17.1 vs. 67.8 ± 16.0) (*p* = 0.036), and M6 (78.6 ± 16.6 vs. 68.2 ± 17.0) (*p* = 0.014) were higher in RTC group than in UC group. Meanwhile, QLQ-C30 global health status score was gradually increased from M0 to M6 in RTC group (*p* < 0.001) and UC group (*p* < 0.001) (Figure 5A). QLQ-C30 function score at M4 (74.5 ± 15.9 vs. 66.0 ± 13.5) (*p* = 0.014) and M6 (76.5 ± 14.4 vs. 67.9 ± 15.4) (*p* = 0.021) were also higher in RTC group than in UC group. Moreover, QLQ-C30 function score was gradually elevated from M0 to M6 in RTC group (*p* < 0.001) and UC group (*p* < 0.001) (Figure 5B). Regarding QLQ-C30 symptoms score, it at M2 (28.6 ± 14.9 vs. 36.1 ± 18.5) (*p* = 0.041) and M4 (26.8 ± 14.3 vs. 34.8 ± 17.7) (*p* = 0.035) were lower in RTC group than in UC group. Furthermore, QLQ-C30 symptoms score was gradually declined from M0 to M6 in RTC group (*p* < 0.001) and UC group (*p* < 0.001) (Figure 5C).

### 3.5. Subgroup analysis of HADS scores and QLQ-C30 scores at M6

In GC patients with recurrence<3 years, HADS-A score (*p* = 0.013) declined while QLQ-C30 global health status score (*p* = 0.017) ascended in RTC group contrasted to UC group. Additionally, in GC patients with recurrence of 3–5 years, there was no discrepancy of HADS scores and QLQ-C30 scores between groups (all *p* > 0.05). In GC patients with recurrence≥5 years, HADS-A score (*p* = 0.032) and HADS-D score (*p* = 0.009) both declined in RTC group contrasted to UC group.

As far as distance metastases at recurrence is concerned, in GC patients without distance metastases at recurrence, only HADS-A score reduced in RTC group contrasted to UC group (*p* = 0.009). In GC patients with distance metastases at recurrence, QLQ-C30 function score ascended (*p* = 0.013) in RTC group contrasted to UC group.

Additionally, in GC patients without radiotherapy, HADS-A score (*p* = 0.001) declined, while QLQ-C30 global health status score (*p* = 0.026) and QLQ-C30 function score (*p* = 0.014) increased in RTC group contrasted to UC group. Moreover, in GC patients with radiotherapy, there was no discrepancy of HADS-scores or QLQ-C30 scores between groups (all *p* > 0.05).

In GC patients without targeted drug therapy, HADS-A score (*p* = 0.034) descended in RTC group contrasted to UC group. In those with targeted drug therapy, HADS-A score (*p* = 0.041) and HADS-D score (*p* = 0.041) both declined, whereas QLQ-C30 global health status score (*p* = 0.006) and QLQ-C30 function score (*p* = 0.012) elevated in RTC group contrasted to UC group.

Furthermore, in GC patients without ICI treatment, HADS-A score (*p* = 0.024) descended but QLQ-C30 global health status score (*p* = 0.007) elevated in RTC group contrasted to UC group. However, in GC patients with ICI treatment, there was no difference in HADS-scores or QLQ-C30 scores between groups (all *p* > 0.05) (Table 2).

**Table 2:** Subgroup analysis of HADS-A, HADS-D, and QLQ-C30 scores at M6 between UC group and RTC group.

Items	Assessment at M6	UC group	RTC group	*p* value
*Time to recurrence*				
<3 years	HADS-A score	8.3 ± 2.8	5.1 ± 2.3	0.013
	HADS-D score	6.6 ± 3.4	4.9 ± 1.6	0.170
	QLQ-C30 global health status score	65.1 ± 18.5	83.4 ± 11.1	0.017
	QLQ-C30 function score	65.0 ± 17.0	77.1 ± 13.0	0.096
	QLQ-C30 symptoms score	25.8 ± 16.0	22.3 ± 13.2	0.608
3–5 years	HADS-A score	6.2 ± 1.9	6.2 ± 2.2	0.988
	HADS-D score	5.3 ± 2.0	5.2 ± 2.5	0.983
	QLQ-C30 global health status score	74.2 ± 11.4	75.7 ± 20.3	0.821
	QLQ-C30 function score	73.5 ± 10.3	75.1 ± 15.1	0.765
	QLQ-C30 symptoms score	26.1 ± 12.7	27.2 ± 14.9	0.839
≥5 years	HADS-A score	7.4 ± 2.7	5.1 ± 1.9	0.032
	HADS-D score	7.4 ± 1.4	4.7 ± 2.6	0.009
	QLQ-C30 global health status score	64.6 ± 20.0	77.9 ± 16.0	0.102
	QLQ-C30 function score	64.6 ± 17.7	77.6 ± 16.0	0.084
	QLQ-C30 symptoms score	37.0 ± 17.8	24.2 ± 15.9	0.089
*Distance metastases at recurrence*				
No	HADS-A score	7.2 ± 2.4	5.4 ± 1.9	0.009
	HADS-D score	5.8 ± 2.0	4.6 ± 2.2	0.074
	QLQ-C30 global health status score	71.6 ± 16.1	79.8 ± 16.7	0.108
	QLQ-C30 function score	72.2 ± 14.2	77.6 ± 16.0	0.248
	QLQ-C30 symptoms score	27.6 ± 14.8	23.2 ± 15.6	0.353
Yes	HADS-A score	7.3 ± 2.9	5.8 ± 2.5	0.185
	HADS-D score	7.5 ± 3.0	5.6 ± 2.4	0.099
	QLQ-C30 global health status score	61.9 ± 17.3	76.4 ± 16.9	0.050
	QLQ-C30 function score	59.9 ± 14.8	74.6 ± 11.7	0.013
	QLQ-C30 symptoms score	33.2 ± 18.0	27.8 ± 12.4	0.408
*Radiotherapy*				
No	HADS-A score	7.7 ± 2.5	5.1 ± 2.1	0.001
	HADS-D score	6.4 ± 2.0	5.1 ± 2.4	0.073
	QLQ-C30 global health status score	67.5 ± 15.8	79.2 ± 16.9	0.026
	QLQ-C30 function score	66.5 ± 13.1	77.6 ± 15.0	0.014
	QLQ-C30 symptoms score	27.5 ± 12.0	23.8 ± 14.5	0.383
Yes	HADS-A score	6.8 ± 2.7	6.6 ± 1.9	0.915
	HADS-D score	6.4 ± 3.1	4.6 ± 2.0	0.127
	QLQ-C30 global health status score	68.9 ± 18.9	76.9 ± 16.6	0.308
	QLQ-C30 function score	69.5 ± 18.2	73.1 ± 13.0	0.612
	QLQ-C30 symptoms score	32.1 ± 10.1	27.7 ± 14.9	0.507
*Targeted drug therapy*				
No	HADS-A score	7.8 ± 2.0	5.6 ± 2.5	0.034
	HADS-D score	6.5 ± 2.0	5.4 ± 2.3	0.244
	QLQ-C30 global health status score	75.8 ± 14.2	77.5 ± 18.7	0.823
	QLQ-C30 function score	71.1 ± 15.6	73.1 ± 13.7	0.759
	QLQ-C30 symptoms score	28.0 ± 15.4	24.0 ± 13.6	0.523
Yes	HADS-A score	7.0 ± 2.8	5.5 ± 2.0	0.041
	HADS-D score	6.3 ± 2.7	4.8 ± 2.3	0.041
	QLQ-C30 global health status score	65.0 ± 17.3	79.1 ± 15.9	0.006
	QLQ-C30 function score	66.5 ± 15.4	78.2 ± 14.8	0.012
	QLQ-C30 symptoms score	30.2 ± 16.5	25.3 ± 15.2	0.307
*ICI treatment*				
No	HADS-A score	7.0 ± 2.6	5.5 ± 1.9	0.024
	HADS-D score	6.0 ± 2.3	4.9 ± 2.3	0.092
	QLQ-C30 global health status score	69.5 ± 16.2	81.5 ± 14.9	0.007
	QLQ-C30 function score	71.0 ± 13.6	78.0 ± 14.6	0.077
	QLQ-C30 symptoms score	28.3 ± 15.8	22.3 ± 14.7	0.160
Yes	HADS-A score	8.1 ± 2.2	5.7 ± 2.9	0.100
	HADS-D score	7.8 ± 2.8	5.3 ± 2.5	0.118
	QLQ-C30 global health status score	63.8 ± 19.8	65.3 ± 18.6	0.882
	QLQ-C30 function score	57.6 ± 17.3	69.7 ± 12.6	0.176
	QLQ-C30 symptoms score	33.6 ± 17.0	36.5 ± 4.8	0.697

UC, usual care program; RTC, reminiscence therapy-based care program; ICI, immune check-point inhibitor; HADS-A, Hospital Anxiety and Depression Scale-anxiety; HADS-D, Hospital Anxiety and Depression Scale-depression; QLQ-C30, Quality of Life Questionnaire-Core 30; SD, standard deviation. All data are presented as mean ± SD.

### 3.6. Subgroup analysis of HADS scores and QLQ-C30 scores in patients with/without baseline anxiety/depression

In GC patients without anxiety at M0, HADS-A score reduced at M6 (*p* = 0.018) in RTC group compared to UC group, however, there was no discrepancy of HADS-D score or QLQ-C30 scores between groups (all *p* > 0.05). Moreover, in GC patients with anxiety at M0, HADS-A score decreased at M2 (*p* = 0.041) and M4 (*p* = 0.026), QLQ-C30 global health status score ascended at M2 (*p* = 0.025), moreover, QLQ-C30 function score increased at M4 (*p* = 0.021) and M6 (*p* = 0.032), while QLQ-C30 symptoms score declined at M2 (*p* = 0.036) in RTC group compared with UC group.

In GC patients without depression at M0, only HADS-A score reduced at M6 (*p* = 0.014) in RTC group contrasted to UC group. Furthermore, in GC patients with depression at M0, HADS-D score descended at M1 (*p* = 0.015) and M6 (*p* = 0.001); notably, QLQ-C30 global health status score and QLQ-C30 function score ascended while QLQ-C30 symptoms score declined at M1, M2, M4, and M6 in RTC group compared with UC group (all *p* < 0.05) (Table 3).

**Table 3:** Subgroup analysis of HADS-A, HADS-D, and QLQ-C30 scores at each assessment time point between UC group and RTC group.

Assessment	Time	UC group	RTC group	*p* value
*Without anxiety at M0*				
HADS-A score	M0	5.6 ± 1.2	5.7 ± 1.4	0.786
	M1	6.2 ± 3.1	6.0 ± 1.6	0.826
	M2	5.8 ± 1.8	6.0 ± 1.9	0.707
	M4	5.9 ± 2.1	5.8 ± 2.0	0.838
	M6	6.8 ± 1.6	5.1 ± 1.7	0.018
HADS-D score	M0	6.2 ± 2.6	6.5 ± 2.4	0.612
	M1	6.1 ± 2.2	6.2 ± 2.5	0.927
	M2	5.2 ± 2.0	5.6 ± 1.2	0.519
	M4	5.6 ± 2.7	5.4 ± 2.5	0.844
	M6	5.8 ± 2.1	4.5 ± 2.6	0.190
QLQ-C30 global health status score	M0	62.4 ± 19.8	61.4 ± 14.3	0.849
	M1	64.8 ± 15.0	67.7 ± 15.4	0.537
	M2	68.9 ± 13.2	70.7 ± 14.7	0.683
	M4	69.6 ± 15.2	75.0 ± 15.8	0.341
	M6	68.9 ± 16.8	79.1 ± 15.3	0.118
QLQ-C30 function score	M0	57.4 ± 18.2	56.1 ± 18.1	0.817
	M1	61.7 ± 16.2	60.8 ± 18.6	0.873
	M2	64.6 ± 13.9	69.0 ± 16.2	0.370
	M4	68.0 ± 13.2	73.3 ± 16.1	0.323
	M6	68.0 ± 14.9	74.1 ± 14.8	0.320
QLQ-C30 symptoms score	M0	38.6 ± 18.4	41.3 ± 16.1	0.607
	M1	35.7 ± 18.3	36.6 ± 14.3	0.850
	M2	32.8 ± 16.9	29.8 ± 15.8	0.565
	M4	31.9 ± 15.7	27.6 ± 14.7	0.432
	M6	25.5 ± 13.3	25.9 ± 14.8	0.935
*With anxiety at M0*				
HADS-A score	M0	10.9 ± 2.6	10.6 ± 2.6	0.711
	M1	9.0 ± 2.6	8.3 ± 2.6	0.332
	M2	8.4 ± 3.0	6.7 ± 2.4	0.041
	M4	8.0 ± 2.6	6.4 ± 2.0	0.026
	M6	7.4 ± 2.9	5.8 ± 2.4	0.056
HADS-D score	M0	8.6 ± 2.7	8.4 ± 2.8	0.804
	M1	8.1 ± 3.0	7.2 ± 2.2	0.249
	M2	7.7 ± 2.8	6.5 ± 2.6	0.151
	M4	7.0 ± 2.8	5.8 ± 2.4	0.174
	M6	6.7 ± 2.6	5.3 ± 2.0	0.076
QLQ-C30 global health status score	M0	58.0 ± 15.6	62.1 ± 15.8	0.358
	M1	60.7 ± 16.4	69.8 ± 16.6	0.055
	M2	64.4 ± 13.4	73.7 ± 13.8	0.025
	M4	66.7 ± 16.6	76.8 ± 18.6	0.067
	M6	67.8 ± 17.8	78.2 ± 17.9	0.068
QLQ-C30 function score	M0	53.8 ± 17.6	58.3 ± 15.4	0.325
	M1	56.5 ± 18.3	63.8 ± 18.1	0.153
	M2	64.5 ± 13.3	71.5 ± 17.3	0.123
	M4	64.8 ± 13.8	75.7 ± 16.1	0.021
	M6	67.8 ± 15.9	78.3 ± 14.3	0.032
QLQ-C30 symptoms score	M0	44.9 ± 19.2	41.0 ± 15.8	0.447
	M1	44.4 ± 20.4	37.5 ± 15.2	0.176
	M2	38.4 ± 19.4	27.4 ± 14.2	0.036
	M4	36.5 ± 18.8	26.1 ± 14.2	0.052
	M6	31.5 ± 17.0	24.1 ± 14.6	0.144
*Without depression at M0*				
HADS-A score	M0	7.3 ± 2.7	6.9 ± 2.6	0.564
	M1	7.0 ± 3.3	6.6 ± 2.4	0.627
	M2	6.4 ± 2.5	5.9 ± 2.2	0.462
	M4	6.7 ± 2.2	5.7 ± 2.2	0.143
	M6	7.1 ± 2.3	5.3 ± 2.1	0.014
HADS-D score	M0	5.5 ± 1.6	5.6 ± 1.5	0.929
	M1	5.5 ± 1.5	6.1 ± 2.2	0.240
	M2	5.3 ± 2.4	5.5 ± 2.1	0.751
	M4	5.5 ± 2.3	5.6 ± 2.5	0.925
	M6	4.9 ± 1.7	5.2 ± 2.3	0.694
QLQ-C30 global health status score	M0	62.4 ± 18.2	62.7 ± 15.5	0.935
	M1	66.8 ± 14.9	68.6 ± 16.0	0.681
	M2	70.8 ± 12.2	72.0 ± 14.3	0.737
	M4	72.9 ± 15.1	76.6 ± 16.5	0.455
	M6	75.9 ± 15.0	78.4 ± 15.3	0.619
QLQ-C30 function score	M0	61.0 ± 17.0	57.4 ± 17.6	0.454
	M1	64.4 ± 15.6	61.2 ± 18.1	0.509
	M2	69.2 ± 14.1	68.7 ± 16.9	0.912
	M4	70.8 ± 13.4	73.0 ± 16.0	0.645
	M6	74.8 ± 15.6	76.0 ± 14.0	0.821
QLQ-C30 symptoms score	M0	35.7 ± 18.4	40.9 ± 16.5	0.290
	M1	33.5 ± 18.6	37.3 ± 14.6	0.416
	M2	29.9 ± 17.1	29.3 ± 16.0	0.910
	M4	28.2 ± 15.4	27.8 ± 15.2	0.935
	M6	22.9 ± 12.8	26.7 ± 15.2	0.433
*With depression at M0*				
HADS-A score	M0	10.4 ± 3.4	9.7 ± 3.3	0.530
	M1	8.9 ± 2.6	7.9 ± 2.3	0.184
	M2	8.5 ± 3.0	6.9 ± 2.1	0.067
	M4	7.9 ± 2.9	6.6 ± 1.5	0.134
	M6	7.4 ± 2.9	5.9 ± 2.2	0.123
HADS-D score	M0	10.0 ± 2.2	9.7 ± 2.1	0.676
	M1	9.4 ± 2.6	7.4 ± 2.5	0.015
	M2	8.3 ± 2.4	6.7 ± 2.7	0.054
	M4	7.5 ± 2.9	5.7 ± 2.5	0.063
	M6	7.8 ± 2.3	4.6 ± 2.3	0.001
QLQ-C30 global health status score	M0	56.9 ± 16.3	60.6 ± 14.4	0.432
	M1	57.2 ± 15.7	68.9 ± 16.1	0.019
	M2	60.9 ± 13.0	72.3 ± 14.4	0.013
	M4	62.1 ± 15.4	75.1 ± 18.4	0.032
	M6	60.4 ± 15.6	78.7 ± 18.7	0.006
QLQ-C30 function score	M0	48.5 ± 16.4	57.0 ± 15.8	0.086
	M1	51.9 ± 17.5	63.6 ± 18.8	0.038
	M2	59.0 ± 10.3	72.2 ± 16.5	0.005
	M4	60.6 ± 11.6	76.6 ± 15.9	0.002
	M6	60.9 ± 11.8	77.2 ± 15.6	0.002
QLQ-C30 symptoms score	M0	50.0 ± 16.8	41.6 ± 15.3	0.088
	M1	49.3 ± 18.1	36.7 ± 13.8	0.015
	M2	43.6 ± 17.6	27.7 ± 13.7	0.003
	M4	42.1 ± 17.5	25.4 ± 13.3	0.004
	M6	36.1 ± 16.4	22.4 ± 13.7	0.019

UC, usual care program; RTC, reminiscence therapy-based care program; HADS-A, Hospital Anxiety and Depression Scale-anxiety; HADS-D, Hospital Anxiety and Depression Scale-depression; QLQ-C30, Quality of Life Questionnaire-Core 30; SD, standard deviation. All data are presented as mean ± SD.

## 4. Discussion

Recurrent GC patients face the dual pressure including physical pain and economic burden, who usually have high incidence rates of anxiety and depression (Zhang, 2021). Therefore, it is curial to find effective managements to relieve anxiety and depression of recurrent GC patients. It is reported that RT alleviates the mental health of some cancer patients (Chen et al., 2022; Liu et al., 2022). For example, one previous study shows that compared with UC, RT involved care program relieve anxiety and depression in postoperative patients with cervical cancer (Liu et al., 2022). Moreover, another study also indicates that care program containing RT is a potential care program to improve mental health in older papillary thyroid carcinoma patients (Chen et al., 2022). However, the influence of RT in recurrent GC patients has been unreported. Our study revealed that RTC reduced HADS scores and anxiety severity in recurrent GC patients compared with UC. This might be because: (1) RTC reviewed past experiences and feelings to arouse the sense of happiness of patients, and established their confidences in resisting diseases, thus relieved their anxiety and depression (Syed Elias et al., 2015; Zhang et al., 2017) and (2) RTC enhanced the patients’ desire to communicate through listening and sharing, alleviating their loneliness and other negative emotions, thus relieved their anxiety and depression (Huang et al., 2022). In addition, anxiety rate showed a decreasing trend at M6 in RTC group compared with UC group, although it did not reach statistical significance. This might because the sample size in this study was small, meanwhile, the statistical effect of the Chi-square test to compare variables between groups was low, resulting in no difference between groups.

The quality of life is as important as the mental health in cancer patients. Due to long-term treatment and loss of physical function, recurrent GC patients generally have poor qualities of life (Kim et al., 2019; Lewandowska et al., 2020). According to previous studies, RT also effectively improves the patients’ quality of life. For example, compared to UC, RT involved care program enhanced quality of life in postoperative patients with cervical cancer (Liu et al., 2022). This was similar to our research, which revealed that RTC improved the quality of life in recurrent GC patients. Possible explanations were as follows: (1) As mentioned above, RTC alleviated anxiety and depression, which might directly relieve the psychological burden of recurrent GC patients, making them face life positively, and thus improving their quality of life (Huang et al., 2022) and (2) RTC strengthened the communications among patients, making them to encourage each other and treat actively, and thus enhanced their quality of life (Li et al., 2022).

Additionally, the subgroup analysis found that RTC was more effective in recurrent GC patients with anxiety or depression at M0. The possible reasons were as follows: (1) Compared with patients without anxiety or depression at M0, recurrent GC patients with anxiety or depression at M0 had increased emotional variability and reduced emotional clarity; therefore, their emotional fluctuations were more intense, and their cognition of emotions were vaguer (Thompson et al., 2017). When treated with RTC, these patients were more likely to be touched by past experience and improve their cognition of emotion through communications, directly regulate emotional response, so as to achieve better treatment effect (Zhang et al., 2017) and (2) RTC could vent patients’ negative emotions by sharing warm memories. Recurrent GC patients with anxiety or depression at M0 were more likely to be moved by these memories and vent negative emotions in time, so the treatment efficacy of RTC in these patients was better (Lazar et al., 2014; Chen et al., 2021). Furthermore, our study also revealed that RTC was more effective in patients without radiotherapy or ICI treatment and patients with targeted drug therapy. However, these findings needed further exploration.

It is worth noting that previous studies have shown that the effect of RTC on alleviating depressive symptoms is persistent (Chiang et al., 2010; Viguer et al., 2017). This was partly similar to our study, which revealed that RTC intervention presented a sustained effect on relieving anxiety and depression, as well as improving the quality of life in recurrent GC patients. This might be because: (1) RTC helped patients increase positive emotions and made them willing to rely on their own abilities to face disease, gradually reducing dependence and adapting to life, increasing social contact, and potentially receiving sustained benefits (Liu et al., 2021; Zhao, 2021) and (2) RTC helped patients develop good habits during the intervention period, making them accustomed to recalling and sharing good memories, thus resulting in sustained benefits (Liu et al., 2021; Cammisuli et al., 2022).

The current study existed some limitations: (1) Our study had a small sample size, and further study should include more recurrent GC patients to verify the outcome of RTC on anxiety, depression, and quality of life, (2) The intervention period was relatively short, and a longer-term intervention was required to appraise the effect of long-term RTC on anxiety, depression, and quality of life in recurrent GC patients, and (3) Our study only evaluated anxiety and depression by HADS, and future studies should use multiple assessment scales for investigation.

In summary, RTC is an effective intervention that relieves anxiety and depression, and enhances quality of life in recurrent GC patients. In clinical practice, RTC can be used as a non-drug intervention to alleviate mental health and improve quality of life in recurrent GC patients. However, future studies with a larger sample size, a longer-term intervention, and multiple assessment scales are required to further confirm the effect of RTC in recurrent GC patients.

## Data availability statement

The original contributions presented in the study are included in the article/supplementary material, further inquiries can be directed to the corresponding author.

## Ethics statement

The studies involving human participants were reviewed and approved by Institution Review Board of HanDan Central Hospital. The patients/participants provided their written informed consent to participate in this study.

## Author contributions

WZ contributed to the conception and the design of the study. XW was responsible for the acquisition, analysis and interpretation of the data. XW and WZ contributed to manuscript drafting or critical revisions of the intellectual content. All authors contributed to the article and approved the submitted version.

## Conflict of interest

The authors declare that the research was conducted in the absence of any commercial or financial relationships that could be construed as a potential conflict of interest.

## Publisher’s note

All claims expressed in this article are solely those of the authors and do not necessarily represent those of their affiliated organizations, or those of the publisher, the editors and the reviewers. Any product that may be evaluated in this article, or claim that may be made by its manufacturer, is not guaranteed or endorsed by the publisher.
